# Geolocator deployment reduces return rate, alters selection, and impacts demography in a small songbird

**DOI:** 10.1371/journal.pone.0207783

**Published:** 2018-12-12

**Authors:** Conor C. Taff, Corey R. Freeman-Gallant, Henry M. Streby, Gunnar R. Kramer

**Affiliations:** 1 Lab of Ornithology and Department of Ecology & Evolutionary Biology, Cornell University, Ithaca, NY, United States of America; 2 Department of Biology, Skidmore College, Saratoga Springs, NY, United States of America; 3 Department of Environmental Sciences, University of Toledo, Toledo, OH, United States of America; Norsk Polarinstitutt, NORWAY

## Abstract

In the past few years, miniature light-level geolocators have been developed for tracking wild bird species that were previously too small to track during their full annual cycle. Geolocators offer an exciting opportunity to study the full annual cycle for many species. However, the potential detrimental effects of carrying geolocators are still poorly understood, especially for small-bodied birds. Here, we deployed light-level geolocators on common yellowthroat warblers (*Geothlypis trichas*). Over two years, we monitored return rates and neighborhood demography for 40 warblers carrying a geolocator and 20 reference birds that did not carry a geolocator. We compared the two groups with long-term data from 108 unmanipulated birds breeding at the same location in previous and subsequent years. Overall, we found that individuals carrying a geolocator were less likely to return to the study site in the following year (21% to 33% returned, depending on inclusion criteria) than either contemporaneous controls (55%) or long-term controls (55%). Among birds marked with geolocators, we also detected viability selection for greater wing length, whereas this pattern was not present in control birds. Finally, in each year after geolocator deployment, inexperienced breeders colonized vacant territories and this demographic effect persisted for two years after deployment. Sexual selection and ornamentation are strongly age-dependent in this system, and behavioral data collected after geolocator deployment is likely to differ systematically from natural conditions. Clearly geolocators will continue to be useful tools, but we suggest that future studies should carefully consider the potential for biased returns and the ecological validity of behavioral data collected from geolocator marked populations.

## Introduction

Light-level geolocators and other miniaturized tracking devices offer an exciting opportunity to gain insight into aspects of the biology of small birds that have been difficult or impossible to study in the past [1]. Geolocators have been increasingly used to describe patterns of migratory connectivity and migratory paths across populations [2–5]. Geolocators have been deployed on wild birds for ten years [2, 6], but within the last five years, the devices have become small enough to allow deployment on species weighing as little as 9 grams. These advances in tag design have led to studies on several species that had previously been studied primarily on the breeding grounds [5, 7–10]. Despite their promise, any information gained by geolocator deployment must be balanced against the possibility of detrimental effects to the animals studied. Although several studies have examined the effects of geolocators on return rates for larger birds [11–14], relatively few have looked at effects on return rates in the smallest birds that can currently be tracked with these devices (but see [7, 9, 15]).

While miniaturized geolocators are relatively new, there is a long tradition of deploying tracking and data logging devices on wild birds and many previous studies have assessed their impact [16, 17]. Meta-analyses have documented pervasive effects of device attachment and suggested that when deploying devices, researchers should consider both the detrimental effects on birds’ performance and the fact that any data recovered may not reflect the typical ecology and behavior of unmanipulated individuals [16, 17]. Geolocators differ from many traditional tracking devices in that they are i) often deployed on smaller birds and ii) designed to stay on for at least one year because the bird must be recaptured to recover the tag with its associated data, whereas many other devices are designed to break away after some period of time [18]. Therefore, it is unclear how similar the effects of geolocator deployment will be to previous work using different devices.

Bridge et al. [11] reviewed the specific effects of geolocator deployment and concluded that no general negative effect on return rate was apparent, but more recent meta-analyses have concluded that geolocators have negative effects on return rate overall [12, 14]. However, these comparative papers only include data on two species under 15 grams and the smallest species included was 12.5 grams. Further miniaturization of tags has allowed recent studies to deploy geolocators on species weighing only 9–15 grams (though it is worth noting that the typical percentage of body weight for tags has remained largely unchanged, [19]). At present, it is unclear whether the patterns identified for larger bodied birds will hold, or be more severe, for birds <15 grams. For example, six species of wood warblers (family Parulidae; 9–14 g) have recently been tracked with geolocators; while a decreased return rate is only reported in two species, two others also had low return rates, but did not include any comparisons with control groups (Table 1). As geolocator technology is adopted and deployed on new species, it is critical that researchers continue to make use of appropriate controls [20]. When possible, this should include both comparisons with long term data for a study population and, most importantly, contemporaneous controls banded in the same year and same conditions as the geolocator deployments to account for year-to-year differences in return rates [21].

**Table 1:** Recent studies using geolocators on small bodied wood warblers (*Parulidae*).

Species	Average mass (g)	Number of geolocators (% return)	Number of controls (% return)	Impact on return rate	Tag percent body mass	Winter range	Citation
Blackpoll Warbler (*Setophaga striata*)	12	37 (16%)	NA	Not reported	4.2%	South America	[5]
Cerulean Warbler (*Setophaga cerulea*)	9	49 (16%)	38 (35%)	Decreased return rate	5.3%	South America	[9]
Connecticut Warbler (*Oporornis agilis*)	13	29 (17%)	NA	Not reported	3.5% (harness not included)	South America	[8]
Common Yellowthroat (*Geothlypis trichas*)	10	40 (33%)	20 (50%)	Decreased return rate (see results)	4.3%	Southern USA, Caribbean, Central America	(this study)
Golden-Winged Warbler (*Vermivora chrysoptera*)	9	40 (46%)	32 (44%)	No effect	5.7% or 5.0% (two models used)	Central or South America	[7]
Kirtland’s Warbler (*Setophaga kirtlandii*)	14	60 (47%) 24 (25%)^a^	32 (56%) 29 (no data)^a^	No effect	4.5% (avg. of two models)	S.E. USA, Caribbean	[32]

^a^ In this study, one group of geolocator and control birds were banded in an earlier year, but only minimal effort was made to re-sight birds the next year, so return estimate is unreliable for geolocator group and not reported for the control group.

In this study, we deployed geolocators on male common yellowthroats (*Geothlypis trichas*) breeding in New York State, USA. Common yellowthroats breed across most of North America and overwinter in the southern USA, Caribbean islands, and Central America [22]; they forage primarily by gleaning insects from leaves and bark and typically remain near the ground or low in dense vegetation [23]. Our population has been studied for >10 years, so ample baseline data were available to compare with that obtained from males carrying geolocators. Moreover, we also compared return rates with that of contemporaneous control birds that were captured in one year that geolocators were deployed, but that did not receive geolocators. We initially asked whether geolocator deployment resulted in decreased return rates when compared with control birds and with the long-term baseline expectations. Next, we asked whether deploying geolocators at a site altered the selection dynamics and local demographic composition in the following year. Specifically, we compared the patterns of viability selection on morphology for geolocator versus control birds and we compared the demographic composition of sites in the year after geolocators were deployed to the long-term baseline values for this population. The second two questions addressed here are critical for assessing the types of studies that can be effectively conducted using geolocators. For some questions—such as where a population overwinters—subtle changes in selection and breeding season demography may be unimportant, but for others—such as whether individual variation in migration behavior predicts breeding success—small changes in selection could limit the ecological validity of geolocator data and potentially lead to spurious conclusions.

## Methods

We studied common yellowthroats at two field sites near Saratoga Springs, New York, USA (43°10′24.6″N, 73°53′19.7″W). Both sites were on land owned by Skidmore College and we had permission to work at each location. All sampling procedures and manipulations were conducted with the approval of the Skidmore College Institutional Animal Care and Use Committee (protocol #134). We captured males between May and July at one site over four consecutive breeding seasons (2015–2018) and at a second, adjacent site over three consecutive breeding seasons (2016–2018). The two sites were separated by dry woodlands unsuitable for yellowthroats, but were <1.5 km apart. We previously studied common yellowthroats breeding at site one from 2005–2012, but only initiated monitoring at site two in 2016; general field methods and site characteristics have been described extensively elsewhere [24, 25]. Briefly, we captured birds using mistnets and playback of male song from a speaker placed in each male’s territory. At the time of capture, we recorded morphological measurements (wing length, tarsus length, and mass) along with a small (~30 μl) blood sample by brachial venipuncture (as in [24]).

In this study, we deployed 20 geolocators on adult males at site one in 2015 and an additional 20 geolocators at site two in 2016 (Biotrack model ML-6 with 5mm light stalk, Wareham, Dorset, UK). To attach the geolocators, we used a modified leg-loop harness [26] as described in Streby et al. [27]. With this harness design, the leg loops are made from elastic cord (Stretch Magic 0.5 mm jewelry cord) and attached to the geolocator ahead of time so that deployment in the field adds only a few minutes to handling time [27]. Peterson et al. [7] previously used the same harness and geolocator model, tested with and without a light stalk, on golden-winged warblers (*Vermivora chrysoptera*; 8–10 grams) and found no effect of the geolocator or the light stalk on return rates. Including the harness, each geolocator weighed approximately 0.45 grams, which was 4.3 ± 0.2% (range 3.9% to 4.7%) of the average body mass of males that received geolocators in this study (mean body mass for geolocator carrying males = 10.46 ± 0.52 g, range = 9.5 to 11.6 g, *n* = 40). We surveyed the study sites the following year to relocate returning birds; all control and geolocator marked birds that were re-encountered were captured and we again collected morphological measurements and a blood sample.

At our sites, males rarely move more than 200 meters between years and the area surrounding the typical yellowthroat territories is composed of woodlands that are too dry for yellowthroats to use [25]. We banded 206 males from 2005–2012 and used the same search strategy described here to detect returns in subsequent years; in only two instances was a male detected again after a failure to detect him in a previous year [28]. From 2015–2018 we banded 124 males and had three additional cases where a male was detected again after a failure to detect him in a previous year (see below). Thus, our detection probability was high, but it is possible that a small number of returning males escaped detection. In 2015 all captured males received geolocators. In 2016 we initiated banding at a new site (hereafter site two) and 20 of 21 males captured at that site received geolocators; in that year, males captured at site one did not receive geolocators and acted as contemporaneous controls (these included 13 newly banded males and 6 recaptured males that had carried geolocators from 2015–16, but were released without tracking devices in 2016 plus the one newly banded control male at site two). Although geolocators were not randomly assigned within each site, the two sites were close together (<1.5 km apart) and males did not differ significantly in pre-treatment mass, wing length, or tarsus length (two sample t-tests, P > 0.1).

We compared the year-to-year return rate of these 20 unmanipulated individuals with that of the 40 geolocator tagged birds. We repeated this comparison with and without the birds that had previously carried geolocators because it was unclear if there would be long-term effects on individuals that had once carried geolocators. To put these results in context, we also examined the year-to-year return rate of 108 additional unmanipulated birds (across 140 breeding records) that were monitored at this study site from 2008 to 2012 and from 2017 to 2018. In addition to looking at differences in return rates, we also asked whether viability selection on morphology differed for control versus geolocator tagged birds and whether the demographic composition of our sites differed in the years after geolocators were deployed.

To evaluate the effects of geolocators on the demographic composition of each site, we grouped males into inexperienced, first time breeders at our site and experienced, returning breeders (as in [24]). Males categorized as inexperienced by this criterion are most likely one-year old [25, 28]; however, we cannot differentiate between one-year old birds and returning breeders that disperse into our site from nearby locations and we therefore refer to these groups as ‘inexperienced’ and ‘experienced’ breeders. Previous work in this population demonstrates that inexperienced and experienced males differ in several indices of condition, plumage coloration and size, patterns of selection on ornamentation, song characteristics, and infection by blood parasites [24, 28–30]. Given these pervasive differences, experimental manipulations that result in a difference in the percentage of inexperienced and experienced males have the potential to obscure or even alter the dynamics of social interaction and selection observed in a local population.

In this study, the experience class of geolocator marked birds in the initial banding year was unknown because we did not capture males at site one in 2013 or 2014 (the location where geolocators were deployed in 2015) or at site two in any year prior to 2016 (the location where geolocators were deployed in 2016). Thus, we could not test for different effects of geolocators in experienced versus inexperienced birds. However, we did capture all males at both sites in the three years (site one) or two years (site two) after geolocator deployment. Using these data, we compared the neighborhood demographic composition in the two or three years after geolocator deployment to the typical demographic composition of this population using banding records from 2008 to 2012.

We compared the return rates of control and geolocator marked birds using odds ratios and Fisher’s exact test. To evaluate differences in the patterns of selection between geolocator and control groups, we performed t-tests comparing the year n morphology (mass, tarsus, and wing length) of birds that did or did not return in the year n + 1 separately for control and geolocator marked individuals. We tested for differences in the demographic composition (ratio of inexperienced to experienced males) in the year after geolocator or control birds were banded using odds ratios and Fisher’s exact test.

Sample sizes vary due to missing data or unknown experience class. All statistics and figures were produced in R version 3.3.3 (R Core Development Team, Vienna, Austria). Odds ratios and associated p-values from contingency tables were calculated using the ‘epitools’ package in R [31]. The data and code used to produce these results have been made available for download as supplementary material (S1 and S2 Files).

## Results

We recaptured 6/20 (30%) geolocator marked birds in 2016 and 5/20 (25%) geolocator marked birds in 2017. Two additional birds tagged in 2016 were not detected on territory in 2017 but were captured in 2018; thus, 7/20 (35%) birds tagged in 2016 eventually returned. Of these thirteen males, six had dropped their geolocator harness during the year (2 in 2016 and 4 in 2017, including the two that were undetected until 2018). Because individuals with lost geolocators carried the units for an indeterminate amount of time, we report results with those individuals both included and excluded. Of the seven units that were recovered, three had physical damage to the waterproof casing and had stopped logging data; we were eventually able to recover >10 months of data from two of these units, but the third yielded no useable data (see discussion). In total, 11/20 of the control birds captured in 2016 were recaptured in subsequent years (one of these was not detected in 2017, but was recaptured in 2018). All individuals that were re-sighted in each year were captured.

### Effect of geolocators on return rates

**Fig 1:**
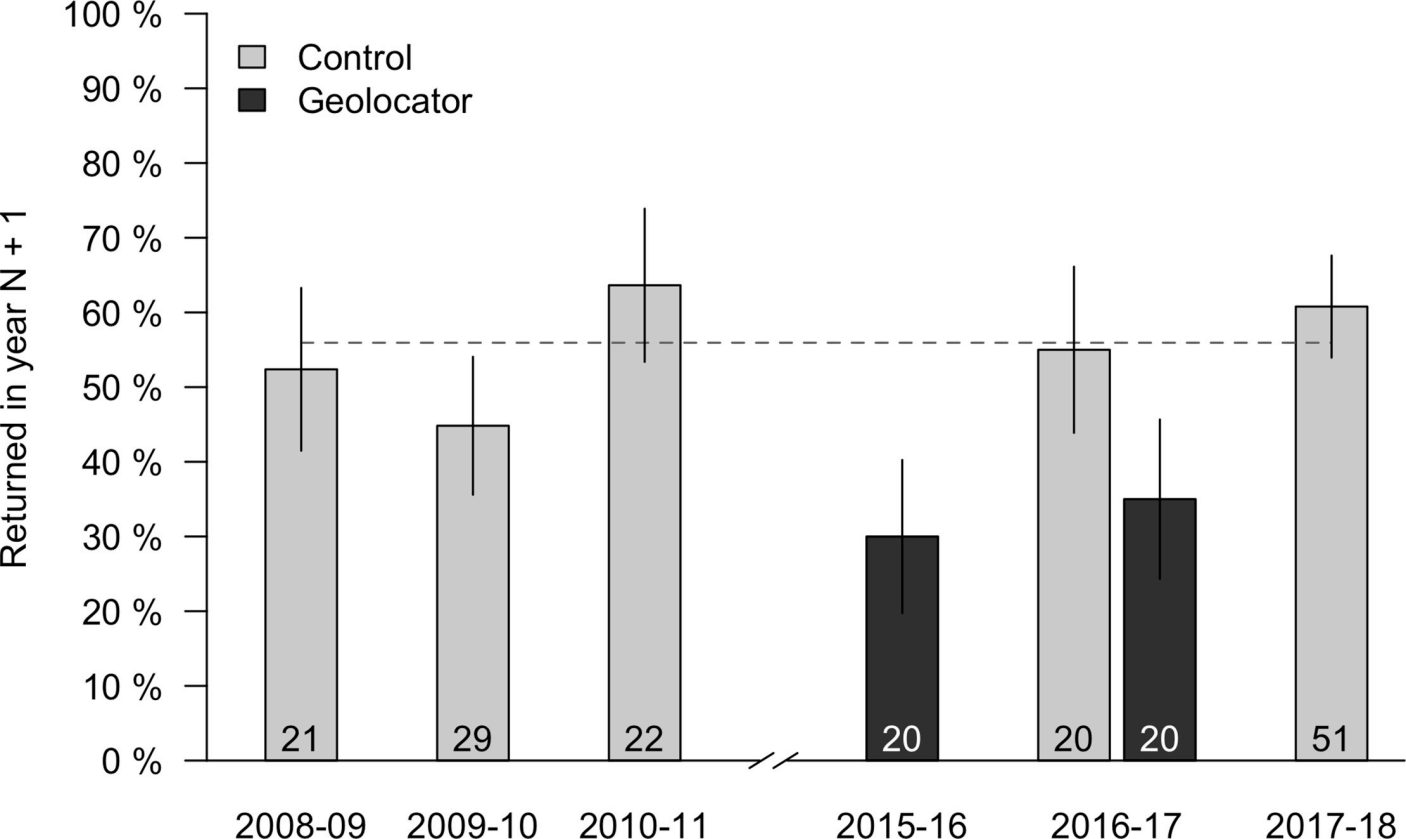
Percent of territory owning males that returned the following year in each of six years. From 2008–2010 and 2017–2018 birds were unmanipulated and represent a baseline survival estimate for this site. In 2015 all territory owners received geolocators. In 2016, banding was expanded to an adjacent site and a mix of control (gray bars) and geolocator tagged (black bars) birds were captured. Error bars indicate binomial standard error and sample size for each group is listed at the bottom of the bar. Dashed gray line indicates the overall mean return rate for control birds when pooling all years.

Overall, the return rates of geolocator marked birds was lower than for control birds. When pooling across all years, 33% of geolocator marked birds returned, but only 21% returned if individuals that dropped their harness are excluded. In contrast, the return rates of control birds from 2016 to 2017 was 55%; when combined with unmanipulated birds banded from 2008 to 2011 and 2017 to 2018, the return rate of control birds was also 55% (*n* = 140 banding records from 108 unique males). Moreover, the return rate for unmanipulated males was similar in all years, but was markedly lower for geolocator marked birds in both years that they were deployed and each of these geolocator years had a lower return rate than any control year (Fig 1; mean return rate for control birds across 5 years = 55%, SD = 7%, range = 45% to 64%).

Combining records from 2015–2016 and 2016–2017, geolocator marked males were significantly less likely to return the following year than control males (odds ratio [OR] for geolocator birds failing to return compared to controls: 0.22, 95% CI: 0.06 to 0.73; n = 54, Fisher’s exact P = 0.02). This result became a non-significant trend when the six individuals that returned with lost geolocators were included (OR: 0.40, CI: 0.13 to 1.22, n = 60, P = 0.10). However, because of the larger sample size, the pattern was significant when including baseline return rate records from 2008–2012 and 2017–2018 regardless of whether the individuals with lost markers were included or not (without lost geolocators: OR: 0.22, CI: 0.08 to 0.51, n = 174, P < 0.001; with lost geolocators: OR: 0.40, CI: 0.18 to 0.83, n = 180, P = 0.02). Irrespective of the significance of the test, the effect size for geolocator application was large; in all comparisons, geolocator tagged males were 60–78% less likely to return when compared to control birds.

### Effect of geolocators on selection

**Fig 2:**
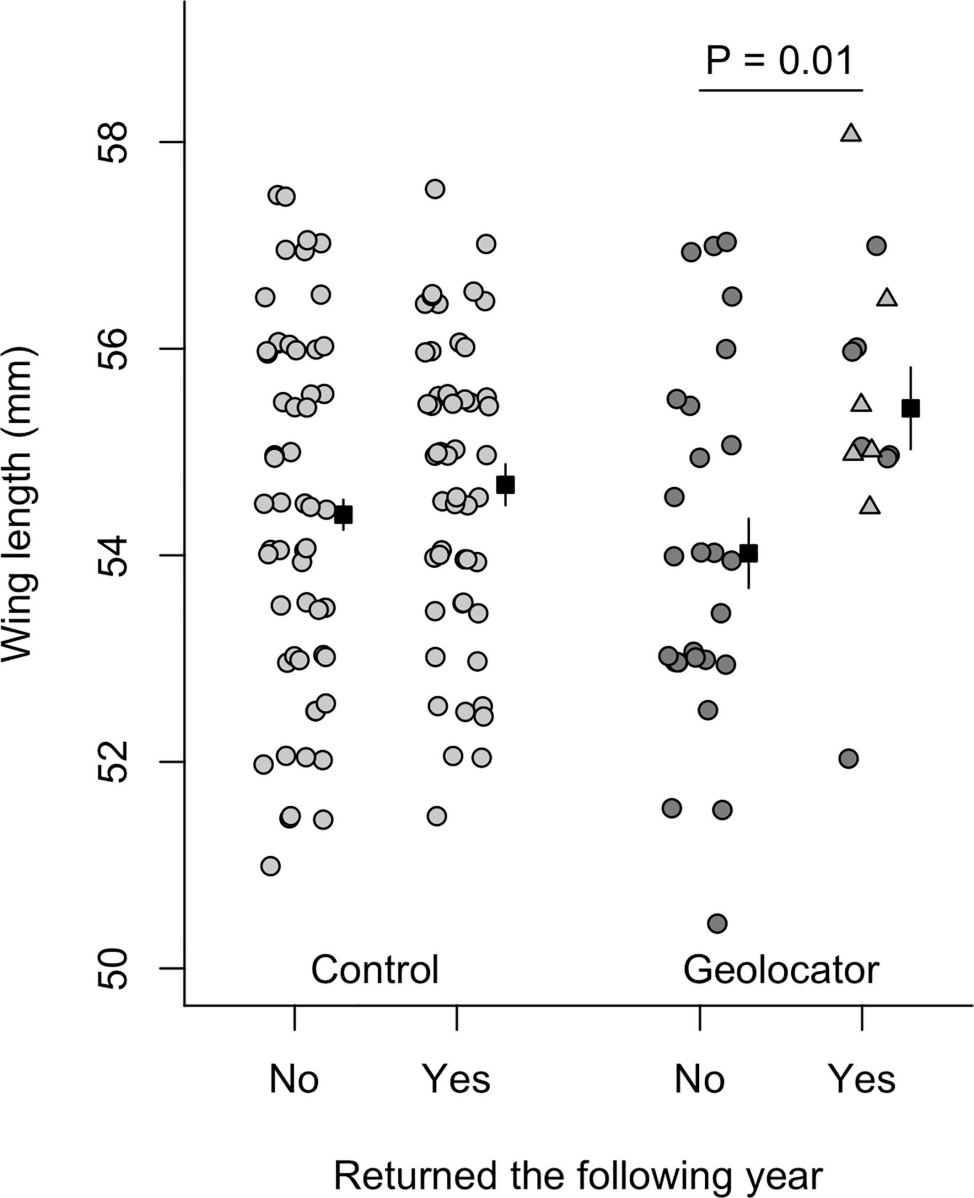
Comparison of wing length for birds that did or did not return to the study site in the year after capture. Data are split by whether or not a geolocator was applied. Raw data points are jittered to improve visibility. For surviving geolocator tagged birds, triangles indicate individuals that returned but had lost their geolocator. Black squares and lines indicate the mean ± SEM for each group.

Mass and tarsus length did not differ between individuals that did or did not return to our site in the subsequent year for either control males or geolocator males (Table 2, P > 0.1). Among geolocator males, individuals with longer wings were significantly more likely to return the following year (Table 2, Fig 2; P = 0.02) whereas wing length did not predict return rates among control males (Table 2, Fig 2). However, the difference in wing length for returning vs. non-returning geolocator tagged birds became non-significant when birds that had dropped their harness were excluded (wing length of returning birds: 55.1 mm, non-returning birds: 54.0, df = 32, t = 1.55, P = 0.13). The effect size in this case was still fairly large (Hedge’s g excluding dropped harness = 0.68, CI: -0.20 to 1.56, including dropped harness = 0.90, CI: 0.18 to 1.61) and the lack of significance was largely due to a reduced sample size. All other comparisons were qualitatively similar regardless of whether birds that had dropped their harness were included or excluded.

**Table 2:** Morphological characteristics in the banding year for males that did or did not return split by whether they received a geolocator or not pooling all years. Confidence intervals that do not overlap 0 are considered significant and shown in bold.

	Control birds				Geolocator birds			
	Return^a^	No Return^a^	n	Hedge’s g	Return^a^	No Return^a^	n	Hedges g
Mass (g)	10.2 ± 0.5	10.3 ± 0.6	137	0.18 [-.16 to .52]	10.4 ± 0.6	10.5 ± 0.5	40	-0.22 [-.90 to .47]
Tarsus (mm)	19.5 ± 0.7	19.4 ± 0.7	139	-0.08 [-.41 to .26]	19.7 ± 0.7	20.0 ± 0.6	40	-0.45 [-1.14 to .24]
Wing (mm)	54.8 ± 1.5	54.4 ± 1.7	140	-0.22 [-.56 to .12]	55.4 ± 1.4	54.0 ± 1.7	40	**0.90** **[.18 to 1.61]**

^a^ Mean ± SD

### Effect of geolocators on demography

**Fig 3:**
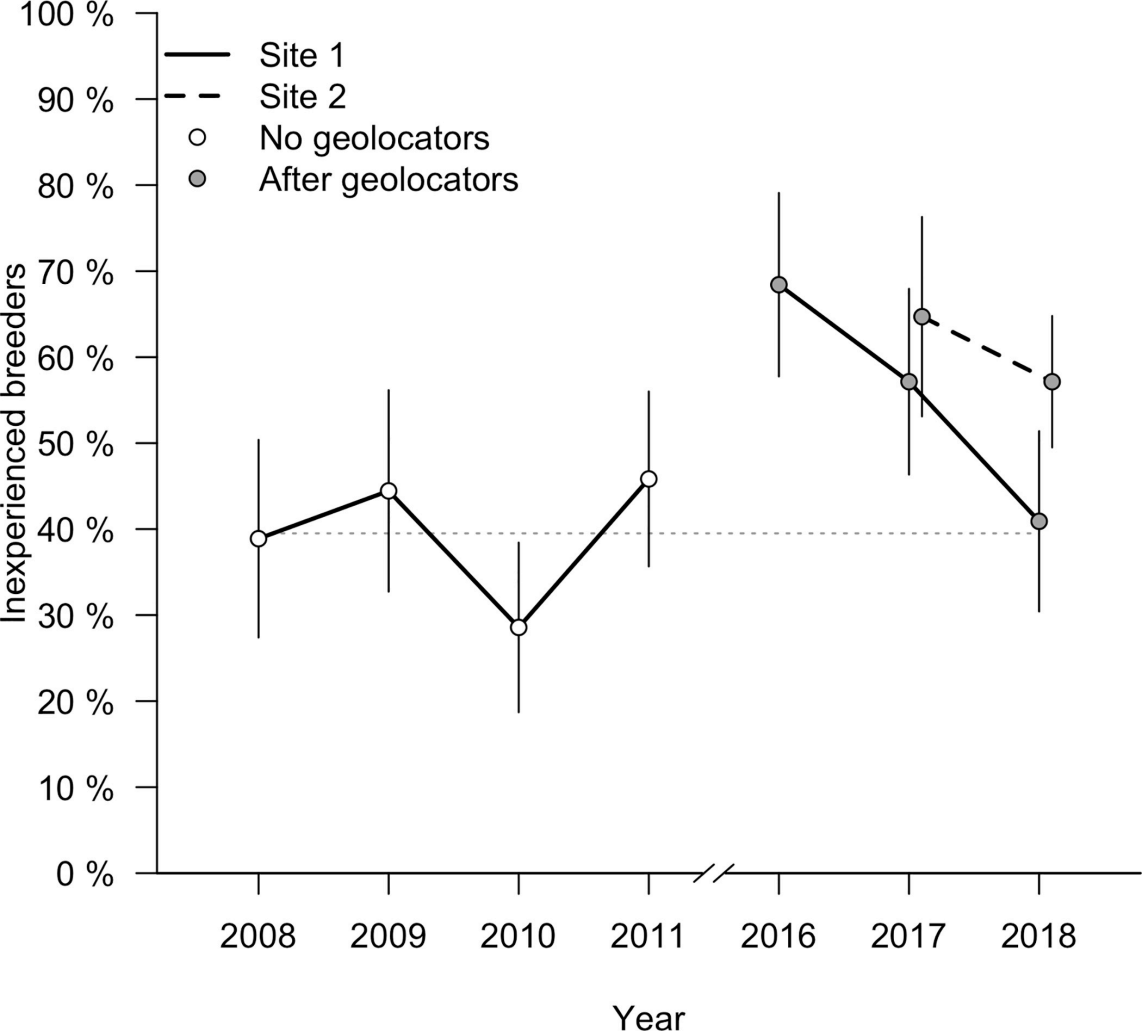
Demographic composition of the field sites in each year of study (percent of territory holders that were inexperienced). Open circles indicate years before any geolocators were deployed. At site 1 (solid line) geolocators were first deployed in 2015 and points illustrate composition one, two, and three years after deployment. At site 2 (dashed line), geolocators were deployed in 2016 and points are one and two years after deployment. Dotted gray line indicates the average percentage of inexperienced males when combining the four pre-geolocator years. Error bars illustrate binomial standard error.

The proportion of inexperienced males breeding at the two sites was higher in the two years after geolocators were deployed when compared to the long-term baseline for this population (Fig 3). Compared to 2008–2011, the year immediately after geolocator deployment—2016 at site one and 2017 at site two—had a significantly higher percentage of inexperienced males (2008–2011 site one: 39.5% inexperienced, one year after deployment at sites one and two: 66.7% inexperienced, OR: 0.33, CI: 0.14 to 0.75, n = 117, Fisher’s exact P < 0.01). Two years after geolocator deployment—2017 at site one and 2018 at site two—the percentage of inexperienced males remained higher than the long-term baseline (two years after deployment combining sites: 57% inexperienced, OR: 0.49, CI: 0.25 to 0.96, n = 144, P = 0.04). Because treatments were deployed at a site level (all geolocators or all controls) and only one or two sites were sampled in each year, we could not separate any possible effect of year or long-term population trends from the effect of geolocator deployment on demography in our analysis. By three years after deployment the percentage of inexperienced males had returned to the long-term baseline at site one (Fig 3; 2018 at site one: 41% inexperienced, OR: 0.94, CI: 0.36 to 2.55, n = 103, P = 1).

## Discussion

Geolocators hold great promise for elucidating the movement and behavior of many species that are too small to carry larger tracking devices and have already proven to be an invaluable tool in collecting data needed for effective conservation and management decisions. However, the effects of carrying geolocators are still unclear for many species. We found that after attaching geolocators to common yellowthroats males were 60–78% less likely to return to our sites the following year. We also documented changes in the patterns of selection on morphology and changes in the percentage of inexperienced breeders in our sites after geolocator deployment. While the information obtained from the geolocators that we recovered is valuable, its utility for some types of questions is limited by the non-random subset of individuals that returned and potentially altered social dynamics.

While there is evidence that geolocators often reduce return rates in broad comparisons across species [12, 14, 17], individual studies report heterogeneous effects and relatively few studies have been conducted on the smallest species that are currently able to carry geolocators. Among projects conducted on species <15 g, recent studies have found both reduced return rates [9] and no effect on return rates [7, 32]. However, several other studies have appeared to find low return rates, but did not specifically test for or report negative impacts of geolocators [5, 8, 33]. In one example, only 3 of 100 geolocators deployed on blackcaps (*Sylvia atricapilla*) were recovered in the following year [10]; the authors attribute this low recovery to unusually cold and wet conditions, but without contemporaneous controls it is impossible to differentiate between the effects of weather and geolocators. Since geolocators small enough for species <15 g have only been available for a few years, it is also possible that currently published studies underestimate detrimental effects if there has been publication bias against studies with few returns. While our results are consistent with a survival cost to geolocator deployment, it is important to note that we cannot rule out the possibility that low return rates were due to permanent or temporary emigration or changes in behavior that reduced detection for geolocator tagged males rather than by reductions in survival.

In addition to reduced return rates, we found that geolocators both imposed selection for longer wing length and preceded an increase in the percentage of inexperienced breeders at a local scale that persisted for two years after deployment. The dynamics of sexual selection and signaling are known to be highly age dependent in this system [24, 25, 28, 29], and any attempt to relate migratory behavior and wintering location data obtained from geolocators to events in the following breeding season risks arriving at spurious conclusions due to the effects of the geolocators themselves. We caution that our experimental design did not allow us to rule out the possibility that some or all of the demographic effect that we observed was driven by yearly differences rather than directly by geolocators; however, the pattern that we observed was consistent across two sites for two years each. Moreover, the fact that site one returned to the long-term baseline three years after geolocator deployment also suggests that our result was not the product of a long-term trend in this population. It is important to note that even in years after geolocator deployment, when return rate was reduced, inexperienced males filled all territories at our field sites. Thus, our data only suggest transient and local effects rather than detrimental effects at a population level; similar transient effects may well be deemed acceptable in many cases in order to obtain the data that geolocators can provide, but they should be considered when evaluating what questions geolocators can effectively answer.

While many studies assess the effect of tracking devices on return rates, few have examined how tagging might alter subsequent behavior and selection; these studies are needed to determine what questions geolocators can effectively address [12]. Many early uses of geolocators focused on describing migration routes, migratory connectivity, and overwintering locations for particular species or populations [2]. These devices also have the potential to provide a powerful way to address classic questions in behavioral ecology that have been difficult to answer directly. For example, how do individual differences in winter departure influence breeding season settlement patterns and breeding success? Our data suggest, however, that geolocators may be less useful for this latter type of question because differential return rates may alter the process that researchers are interested in studying. Even in studies of migration, care should be taken when return rates for geolocator marked birds are related to aspects of the phenotype. In cases where the same phenotype (e.g., wing length) is related to both ability to carry a geolocator and migratory path or wintering location, there is the potential to draw erroneous conclusions from geolocator recoveries. Even for individuals that do manage to return with geolocators, the apparent effects on return rates suggest that there might also be impacts on other aspects of migration strategy, such as departure times, stopover durations, and migration speed [20]. Importantly, the geolocators we used had light stalks, a common feature in similar studies, that can produce aerodynamic drag sufficient to reduce flight ranges by 14% in small songbirds [34]. We cannot rule out the possibility that stalkless units or an alternative tag or harness design would mitigate the effects that we observed. One study with a moderate sample size on another small warbler found no effect of stalks on return rates [7], but larger studies on other species have sometimes found moderate effects of stalk length or stalk presence on return rates [13, 35].

Of the thirteen geolocator tagged birds that we recaptured, six had dropped their harness and an additional three returned with geolocators that were damaged. In all three of these cases, the weatherproofing material on the leading edge of the geolocator had split, allowing water to enter and damage the unit. While dropped harnesses and electronic failures have been regularly reported in geolocator studies (reviewed in [11]), our failure rates seemed unusually high, albeit with a small sample size. Common yellowthroats inhabit marshy habitat and regularly move low to the ground through dense vegetation [22]. We suspect that this behavior might have produced the damage that we observed (i.e., the weatherproofing could have split after repeated contact with vegetation). Similarly, this behavior might explain the relatively high rate of harness loss and could have contributed to lower return rates if the repeated drag of carrying the geolocators was more costly for yellowthroats than for similarly sized species that do not spend so much time moving through vegetation or if frequent contact with vegetation increased the risk of entanglement. Our results suggest that the impacts of geolocators may differ for species with different life history traits, but there is currently not enough information to test this hypothesis fully.

Geolocators clearly have great value in studies addressing the ecology, behavior, and conservation of wild birds. These devices have the potential to collect information that cannot be gained in any other way and to connect processes occurring throughout the annual cycle [1, 2, 36]. However, more attention should be paid to the potential impacts of geolocator use and to the ecological validity of data collected from birds carrying geolocators, even when their effects are sub-lethal. In many cases, a moderate increase in mortality may be deemed acceptable in order to gain critical information on migratory paths and wintering locations, but those decisions should be made with the best information available. Future work should aim to include robust controls that allow for accurate estimation of the impact of geolocators, particularly when new species are studied.

## Supporting information

S1 File Data used to produce analyses presented in this study. The complete dataset used in this study.
(TXT)

S2 File Code used to produce analyses in this study. This R script contains the code necessary to recreate all of the analyses and figures presented in this study using the dataset provided above.
(R) Click here for additional data file.
